# Supplementary material for: Local human impacts disrupt depth-dependent zonation of tropical reef fish communities
Source: Nat Ecol Evol. 2023 Sep 25;7(11):1844–55. doi: 10.1038/s41559-023-02201-x (PMC10627831; doi:10.1038/s41559-023-02201-x)
Supplement: Supplementary file 1 — Supplementary Tables 1–12 and Fig. 1. [file 41559_2023_2201_MOESM1_ESM.pdf]

nature ecology & evolution

Article

https://doi.org/10.1038/s41559-023-02201-x

Local human impacts disrupt depth-dependent zonation of tropical reef fish communities

In the format provided by the authors and unedited

Supplementary Information: Table of contents

Supplementary Table 1 Surveyed island information 2
Supplementary Table 2 Bathymetric and shoreline information 3
Supplementary Table 3 Prior distributions for Bayesian models of reef fish biomass 5
Supplementary Table 4 Coefficient estimates for fish biomass models 6
Supplementary Table 5 Maximum probability of effect estimates for model parameters 7
Supplementary Table 6 Unadjusted marginal and conditional R^2 values 8
Supplementary Table 7 Probability of greater absolute increase in fish biomass at unpopulated versus populated islands across depth 9
Supplementary Table 8 Probability of greater percentage increase in fish biomass at populated versus unpopulated islands across depth 10
Supplementary Table 9 Stationary point count surveys with zero-count observations of trophic groups of fish across depth 11
Supplementary Table 10 Cross-scale variance partitioning among spatial random-effect terms 12
Supplementary Table 11 Probabilities of differing variation in fish biomass across spatial scales 13
Supplementary Table 12 Surveyed fish taxa omitted from the study analyses 14
Supplementary Figure 1 Bivariate correlations between biomass models predictor coefficients 16

Supplementary Table 1 Surveyed island information, including geographic location within ecoregions, latitude (lat) and longitude (long), human population (pop.) status (populated islands, P, highlighted in grey; unpopulated islands, U, in white), human population estimates from the 2010 US census (www.census.gov/2010census), average island bathymetric steepness, sampling effort (stationary point count, SPC, surveys and number of sites), and survey year(s). An online map viewer of all study sites is available here: http://noaa.maps.arcgis.com/apps/webappviewer/index.html?id=da5c18ea60d049588fca5feecb82fe07.

**Island**	**Island code**	**Lat**	**Long**	**Pop. status**	**Human population**	**Mean ± SE bathy. steepness (°)**	**No. of SPC surveys (*n*)**	**No. of sites**	**Years surveyed**
**Mariana Islands**									
Farallon de Pajaros	FDP	20.542	144.895	U	-	20.68 ± 0.69	52	23	2011, 2014
Maug	MAU	20.023	145.222	U	-	21.13 ± 0.39	120	52	2011, 2014
Asuncion	ASU	19.693	145.401	U	-	17.22 ± 0.82	90	41	2011, 2014
Agrihan	AGR	18.775	145.668	U	-	12.31 ± 1.03	46	20	2011
Pagan	PAG	18.105	145.760	U	-	11.04 ± 0.60	170	72	2011, 2014
Alamagan	ALA	17.597	145.832	U	-	14.72 ± 1.75	32	16	2011, 2014
Guguan	GUG	17.312	145.837	U	-	14.84 ± 1.52	48	21	2011, 2014
Sarigan	SAR	16.705	145.774	U	-	18.79 ± 0.81	56	20	2011, 2014
Saipan	SAI	15.191	145.735	P	48,220	7.92 ± 0.29	176	77	2011, 2014
Tinian	TIN	15.025	145.627	P	3,136	8.41 ± 0.43	84	38	2011, 2014
Aguijan	AGU	14.850	145.553	P	-	13.90 ± 0.50	48	23	2011, 2014
Rota	ROT	14.149	145.193	P	2,527	7.51 ± 0.33	104	52	2011, 2014
Guam	GUA	13.451	144.773	P	162,810	9.21 ± 0.21	480	237	2011, 2014
**Hawaii Islands**									
Kure	KUR	28.413	-178.339	U	-	6.71 ± 0.17	86	30	2010, 2012
Pearl & Hermes	PHR	27.876	-175.799	U	-	2.37 ± 0.10	146	47	2010, 2011, 2012
Lisianski	LIS	26.016	-173.941	U	-	1.77 ± 0.06	212	87	2010, 2011, 2012, 2014
French Frigate	FFS	23.767	-166.228	U	-	1.98 ± 0.07	74	28	2010, 2011, 2012, 2014
Niihau	NII	21.917	-160.157	U*	170	1.21 ± 0.09	106	42	2010, 2013
Johnston	JOH	16.746	-169.510	U	-	9.10 ± 0.30	26	11	2010, 2012
Kauai	KAU	22.037	-159.568	P	65,689	1.69 ± 0.06	160	63	2010, 2013
Oahu	OAH	21.471	-157.956	P	953,207	1.60 ± 0.04	338	134	2010, 2012, 2013
Molokai	MOL	21.102	-157.058	P	7,404	2.20 ± 0.07	216	99	2010, 2012, 2013
Lanai	LAN	20.850	-156.900	P	3,102	3.49 ± 0.13	176	74	2010, 2012, 2013
Maui	MAI	20.834	-156.369	P	144,444	2.57 ± 0.08	286	116	2010, 2012, 2013
Hawaii	HAW	19.683	-155.548	P	185,079	10.24 ± 0.65	275	101	2010, 2013
**Line Islands**									
Kingman	KIN	6.405	-162.405	U	-	18.88 ± 0.81	108	43	2010, 2012
Palmyra	PAL	5.877	-162.092	U	20	11.42 ± 0.62	203	79	2010, 2012
Jarvis	JAR	-0.374	-159.993	U	-	19.80 ± 0.93	180	72	2010, 2012
**Phoenix/Tokelau/Northern Cook Islands**									
Howland	HOW	0.807	-176.620	U	-	31.20 ± 0.83	142	55	2010, 2012
Baker	BAK	0.194	-176.474	U	-	16.23 ± 1.05	120	45	2010, 2012
Swains	SWA	-11.056	-171.080	U	17	40.48 ± 0.10	154	62	2010, 2012
**Samoa Islands**									
Ofu & Olosega	O&O	-14.172	-169.649	P	353	8.51 ± 0.27	158	60	2010, 2012
Tau	TAU	-14.240	-169.464	P	790	18.25 ± 0.65	120	46	2010, 2012
Tutuila	TUT	-14.295	-170.681	P	55,149	6.98 ± 0.12	583	210	2010, 2012
Rose	ROS	-14.543	-168.156	U	-	34.37 ± 0.33	150	57	2010, 2012

* Niihau Island was classified as unpopulated despite its human population marginally exceeding the threshold limits (<50 residents) and being located approximately 30 km from neighbouring populated island Kauai. Niihau is privately owned and closed to the public and therefore was considered relatively free from local human impacts.

Supplementary Table 2 Bathymetric and shoreline information sources used to create continuous bathymetric surfaces for each island in the study. Where multiple input bathymetry data sources are listed, they were mosaicked together with priority given to higher resolution datasets (as shown in 'Mosaic Resolution' column), then any remaining data holes were filled by interpolation with the Topo To Raster tool in ArcGIS, with the indicated shoreline dataset (if any) used as an additional input with a specified elevation of 0 m. The mean and standard error (SE) percentage of pixels derived from bathymetric data (as opposed to interpolated values) used to estimate survey site-level bathymetric steepness within 400 m radial buffers of sites at each island, are shown. Available high resolution bathymetry data frequently has data gaps. We created gapless continuous depth surfaces for each island by combining existing bathymetry datasets into mosaics of consistent grid resolution with preference for the highest resolution available with the best area coverage. While imagery derived depths alone often have the highest resolution for a given island, it is typically the least accurate and is limited to 4–5 m depths. Reef zone data provided by NOAA ESD were used to remove land, and interior habitat zones (e.g. lagoon, back reef, reef flat) before summarizing slope values. All analyses were done in the appropriate Universal Transverse Mercator zone for each island.

**Island**	**Island code**	**Mosaic resolution (m)**	**Input bathymetric information sources**	**Mean ± SE % pixels derived from bathy. information sources**	**Input shoreline information**
**Hawaii Islands**					
French Frigate Shoals	FFS	20	5 m MB (PIBHMC) + 20 m MBDB (PIBHMC; IKONOS imagery) + 20 m MB (NCEI AutoGrid)	52.01 ± 5.67	NOAA ESD (islands.shp)
Hawaii	HAW	50	50 m multibeam synthesis (HMRG v19)	77.21 ± 3.92	None (not needed)
Johnston	JOH	20	20 m MB + 5 m MBDB (PIBHMC; IKONOS imagery)	98.18 ± 0.31	Digitized from ESRI World Imagery
Kauai	KAU	50	50 m multibeam synthesis (HMRG v19)	99.21 ± 0.46	None (not needed)
Kure	KUR	5	5 m MBDB (PIBHMC; IKONOS imagery)	98.51 ± 0.44	NOAA CUSP
Lanai	LAN	50	50 m multibeam synthesis (HMRG v19)	67.37 ± 4.81	None (not needed)
Lisianski	LIS	20	20 m MB (PIBHMC) + 4 m DB (NOAA CCMA; IKONOS imagery)	60.94 ± 3.51	NOAA CUSP
Maui	MAI	50	50 m multibeam synthesis (HMRG v19)	91.45 ± 1.61	None (not needed)
Molokai	MOL	50	50 m multibeam synthesis (HMRG v19)	91.13 ± 2.10	None (not needed)
Niihau	NII	50	50 m multibeam synthesis (HMRG v19)	44.97 ± 6.84	None (not needed)
Oahu	OAH	50	50 m multibeam synthesis (HMRG v19)	99.99 ± 0.01	None (not needed)
Pearl & Hermes	PHR	20	All PIBHMC: 5m MBDB (IKONOS imagery) + 20 m MB + 40 m MB	70.49 ± 4.85	None
**Line Islands**					
Jarvis	JAR	20	20 m MB (PIBHMC)	81.43 ± 2.43	NOAA CUSP
Kingman	KIN	20	20 m MB + 5 m MBDB (PIBHMC; IKONOS imagery)	98.04 ± 0.61	NOAA CUSP
Palmyra	PAL	40	40 m MB + 5 m MBDB (PIBHMC; IKONOS imagery)	99.24 ± 0.24	NOAA CUSP
**Mariana Islands**					
Agrihan	AGR	10	10 m MB (PIBHMC)	45.90 ± 5.58	NOAA CUSP
Aguijan	AGU	5	5 m MBDBLD (PIBHMC; IKONOS imagery)	0.00 ± 0.00	NOAA CUSP
Alamagan	ALA	10	10 m MB (PIBHMC)	63.20 ± 4.42	NOAA CUSP
Asuncion	ASC	10	10 m MB (PIBHMC)	69.72 ± 1.30	NOAA CUSP
Farallon de Pajaros	FDP	10	10 m MB (PIBHMC)	81.19 ± 0.93	NOAA CUSP

Guam	GUA	10	10 m NOAA NCEI 1/3 arc-second gap-filled CEM + 5 m MBLD (PIBHMC)	99.79 ± 0.06	None
Guguan	GUG	10	10 m MB (PIBHMC)	71.27 ± 2.00	NOAA CUSP
Maug	MAU	10	10 m MB (PIBHMC)	63.29 ± 1.45	NOAA CUSP
Pagan	PAG	10	10 m MB (PIBHMC)	60.28 ± 2.27	NOAA CUSP
Rota	ROT	5	5 m MBDB (PIBHMC; WV2 imagery)	84.16 ± 3.76	NOAA CUSP
Saipan	SAI	5	5 m MBLD (PIBHMC)	98.45 ± 0.44	NOAA CUSP
Sarigan	SAR	10	10 m MB (PIBHMC)	62.89 ± 3.08	NOAA CUSP
Tinian	TIN	5	5 m MBDBLD (PIBHMC; IKONOS imagery)	98.24 ± 0.56	NOAA CUSP
**Phoenix/Tokelau/Northern Cook Islands**					
Baker	BAK	5	5 m MBDB (PIBHMC; WV2 imagery) + 40 m MB (PIBHMC)	95.83 ± 0.88	NOAA CUSP
Howland	HOW	5	5 m MB + 40m MB (PIBHMC)	85.68 ± 1.97	NOAA CUSP
Swains	SWA	10	10 m MB (PIBHMC) + 40 m MB (PIBHMC)	84.28 ± 0.25	NOAA CUSP
**Samoa Islands**					
Ofu & Olosega	O&O	5	5 m MBDB (PIBHMC; IKONOS imagery) + 20 m (NCEI AutoGrid)	98.68 ± 0.43	NOAA ESD (islands.shp)
Rose	ROS	5	5 m MBDB (PIBHMC; IKONOS imagery) + 40 m MB (PIBHMC)	98.76 ± 0.28	NOAA ESD (islands.shp)
Tau	TAU	5	5 m MBDB (PIBHMC; IKONOS imagery) + 20 m MB (NCEI AutoGrid; cleaned)	89.87 ± 1.63	NOAA ESD (islands.shp)
Tutuila	TUT	10	10 m (NOAA NCEI 1/3 arc-second gap-filled CEM)	99.62 ± 0.04	None

Acronyms: MB = Multibeam SONAR bathymetry; DB = Derived bathymetry from satellite imagery (i.e. IKONOS or Worldview2 (WV2), as specified); LD = bathymetric Lidar Depths; CEM = Coastal Elevation Model (topobathy); CUSP = Continually Updated Shoreline Product; PIBHMC = Pacific Islands Benthic Habitat Mapping Center; HMRG = Hawaii Mapping Research Group; NCEI = National Centers for Environmental Information; CCMA = Center for Coastal Monitoring and Assessment; ESD = Ecosystem Sciences Division (NOAA Pacific Islands Fisheries Science Center)
Note: Strings of consecutive 2 letter acronyms (MB, DB, and LD) in the 'input bathymetric data sources' column indicate mosaic data products available from PIBHMC produced from multiple types of bathymetry sources (e.g. MBDB = a mosaic product of multibeam bathymetry and imagery derived bathymetry).

Supplementary Table 3 Prior distributions for Bayesian models of reef fish biomass. Intercept (α), population effects (β), group-level effects (γ), and Gamma distribution shape (ς). Note, σ_j stands for the standard deviation for random effect *j*, with the exception of the random depth effect within islands (σ DEPTH : ISLAND) and priors for coefficients for the splines for steepness, and steepness by depth.

**Biomass model (g-m^{-2})**	**Parameter**	**Model prior**
Common parameters (positive biomass)	β<br>γ<br>σ_j<br>σ DEPTH : ISLAND<br>σ_{spline}<br>gamma shape ς	*Normal* (0, 2)<br>*Normal* (0, σj)<br>*Cauchy* (0, 5)<br>*Cauchy* (0, 1)<br>*Cauchy* (0, 10)<br>*Gamma* (0.01, 0.01)
Common parameters (hurdle)	α (hurdle)<br>β (hurdle)<br>γ (hurdle)<br>σ_j (hurdle)<br>σ DEPTH : ISLAND (hurdle)<br>σ_{spline} (hurdle)<br>gamma shape ς	*Logistic* (-2, 0.5)<br>*Normal* (0, 2)<br>*Normal* (0, σj)<br>*Cauchy* (0, 5)<br>*Cauchy* (0, 1)<br>*Cauchy* (0, 10)<br>*Gamma* (0.01, 0.01)
Total fish biomass	α	*Normal* (4.6, 1)
Primary consumer	α	*Normal* (4.37, 1)
Planktivore	α (positive biomass)	*Normal* (3.19, 1)
Secondary consumer	α	*Normal* (3.27793, 1)
Piscivore	α (positive biomass)	*Normal* (2.78, 1)

Supplementary Table 4 Coefficient estimates and percentile credible intervals from Bayesian mixed-effects models of reef fish biomass (g-m^2). Population status (p) represents the effect of islands populated by humans versus unpopulated islands. All positive biomass response variables were modelled with a Gamma distribution on the log-scale, and where hurdle models were applied (HU), presence-absence biomass were modelled with a binomial distribution and logit function. Non-linear fitting basis splines (s) in depth-steepness interaction terms are indicated by s1 and s2. Continuous predictor variables were centred and scaled. Resulting model coefficients and 95% and 75% credible intervals are not back transformed.

Response	Predictor	Estimate	95% CI		75% CI	
Total fish biomass	(intercept)	4.09	3.63	4.70	3.83	4.35
	depth	0.11	-0.23	0.45	-0.08	0.31
	population status (p)	-0.85	-1.08	-0.61	-0.99	-0.71
	bathymetric steepness	0.26	-0.05	0.59	0.07	0.46
	depth : population status (p)	0.04	-0.08	0.16	-0.03	0.11
	depth : bathymetric steepness s1	0.05	-3.92	3.71	-2.20	2.33
	depth : bathymetric steepness s2	0.04	-3.81	3.75	-2.31	2.29
Primary consumer	(intercept)	2.97	2.47	3.85	2.66	3.31
	depth	-0.30	-0.70	0.09	-0.54	-0.06
	population status (p)	-0.63	-0.92	-0.33	-0.80	-0.45
	bathymetric steepness	0.28	-0.12	0.68	0.05	0.51
	depth : population status (p)	0.23	0.08	0.39	0.14	0.31
	depth : bathymetric steepness s1	0.05	-3.90	3.77	-2.20	2.27
	depth : bathymetric steepness s2	-0.01	-3.84	3.74	-2.23	2.30
Planktivore	(intercept)	2.25	1.36	3.50	1.69	2.84
	(HU intercept)	-2.95	-5.90	-0.84	-4.29	-1.73
	depth	0.46	0.00	0.89	0.19	0.72
	population status (p)	-0.72	-1.08	-0.38	-0.92	-0.52
	bathymetric steepness	0.54	0.08	0.97	0.28	0.78
	depth : population status (p)	-0.12	-0.38	0.14	-0.26	0.02
	depth : bathymetric steepness s1	0.04	-3.81	3.74	-2.26	2.29
	depth : bathymetric steepness s2	0.02	-3.94	3.88	-2.25	2.25
	HU depth	-1.52	-3.11	0.07	-2.40	-0.62
	HU population status (p)	0.22	-0.78	1.05	-0.29	0.70
	HU bathymetric steepness	-2.46	-4.18	-0.81	-3.41	-1.50
	HU depth : population status (p)	0.42	-0.25	1.15	0.02	0.82
	HU depth : bathymetric steepness s1	-0.21	-4.21	3.72	-2.59	2.15
	HU depth : bathymetric steepness s2	0.00	-3.94	4.08	-2.47	2.36
Secondary consumer	(intercept)	2.25	1.85	2.70	2.03	2.48
	depth	-0.01	-0.36	0.34	-0.21	0.19
	population status (p)	-0.51	-0.72	-0.31	-0.63	-0.40
	bathymetric steepness	0.09	-0.24	0.43	-0.11	0.28
	depth : population status (p)	0.04	-0.07	0.16	-0.03	0.11
	depth : bathymetric steepness s1	0.04	-3.88	3.84	-2.27	2.31
	depth : bathymetric steepness s2	0.02	-3.82	3.89	-2.28	2.35
Piscivore	(intercept)	2.70	2.08	3.52	2.35	3.08
	(HU intercept)	-3.65	-5.06	-2.19	-4.49	-2.82
	depth	0.38	0.02	0.72	0.19	0.58
	population status (p)	-1.61	-1.97	-1.25	-1.82	-1.41
	bathymetric steepness	0.03	-0.32	0.37	-0.17	0.23
	depth : population status (p)	0.08	-0.07	0.23	-0.01	0.16
	depth : bathymetric steepness s1	-0.01	-4.08	3.96	-2.42	2.36
	depth : bathymetric steepness s2	-0.05	-3.93	4.05	-2.20	2.27
	HU depth	-0.47	-1.21	0.31	-0.94	-0.00
	HU population status (p)	1.48	0.76	2.24	1.07	1.90
	HU bathymetric steepness	-1.09	-1.97	-0.28	-1.58	-0.59
	HU depth : population status (p)	0.50	0.03	0.98	0.22	0.77
	HU depth : bathymetric steepness s1	-0.11	-4.05	3.64	-2.46	2.20
	HU depth : bathymetric steepness s2	-0.00	-3.86	3.91	-2.31	2.36

Supplementary Table 5 Maximum probability of effect estimates for specified parameters in fitted mixed-effects models of reef fish biomass. Estimates represent the probability (expressed in percentage) that model parameters are strictly positive or negative, and are calculated as the proportion of the posterior distribution that is of the median's sign. Effect estimates of non-linear fitting basis splines (s) in depth-steepness interaction terms are indicated by s1 and s2. Population status indicates the effect of human 'populated' islands. Probability of direction estimates for hurdle model parameters (HU) are shown where applicable.

**Model parameter**	**Total fish biomass**	**Primary consumer**	**Planktivore**	**Secondary consumer**	**Piscivore**
Depth	**75.10**	**93.35**	**97.60**	51.45	**98.15**
HU depth			**96.75**		**87.75**
Population status (P)	**100.00**	**100.00**	**100.00**	**100.00**	**100.00**
HU population status (P)			71.60		**100.00**
Steepness	**94.75**	**92.00**	**99.10**	69.10	54.50
HU steepness			**99.75**		**99.65**
Depth : population status (P)	74.35	**99.80**	**84.15**	**77.15**	**84.25**
HU depth : population status (P)			**88.65**		**98.10**
Depth : bathymetric steepness s1	51.10	51.60	52.05	50.00	50.20
HU depth : bathymetric steepness s1			54.25		51.70
Depth : bathymetric steepness s2	51.65	50.25	50.80	50.10	51.95
HU depth : bathymetric steepness s2			50.95		50.50

Probability estimates ≥75.00% are highlighted in bold.

Supplementary Table 6 Unadjusted marginal (fixed effects only) and conditional (fixed and random effects) R^2 values and percentiles for fitted Bayesian mixed-effects models of reef fish biomass. Bayesian regression model R^2 is estimated as the predicted values variance divided by the variance of predicted values plus the expected variance of the errors (standard error)[1].

**Response**		**Bayesian R^2**	**95% CI**		**75% CI**	
Total fish biomass	Marginal	0.12	0.02	0.43	0.03	0.21
	Conditional	0.55	0.44	0.67	0.44	0.62
Primary consumer	Marginal	0.29	0.06	0.50	0.14	0.50
	Conditional	0.54	0.51	0.57	0.52	0.55
Planktivore	Marginal	0.05	0.001	0.45	0.001	0.17
	Conditional	0.48	0.24	0.67	0.33	0.64
Secondary consumer	Marginal	0.02	0.003	0.10	0.01	0.04
	Conditional	0.37	0.31	0.47	0.32	0.42
Piscivore	Marginal	0.12	0.02	0.42	0.02	0.22
	Conditional	0.52	0.38	0.62	0.45	0.62

Supplementary Table 7 Probability of greater absolute increase in fish biomass at unpopulated versus populated islands across specified 10-m depth intervals. Probability estimates are derived from the difference in posterior predicted changes in fish biomass at specified depth ranges between unpopulated and populated islands with bathymetric steepness held constant at the study mean value.

**Depth (m)**	**Total fish biomass**	**Primary consumer**	**Planktivore**	**Secondary consumer**	**Piscivore**
0–10	**0.98**	0.05	**1.00**	**0.90**	**1.00**
10–20	**0.83**	0.00	**1.00**	0.60	**1.00**
20–30	0.27	0.00	**0.89**	0.15	**0.76**

Probability estimates ≥75.00% are highlighted in bold.

Supplementary Table 8 Probability of greater percentage (%) increase in fish biomass at populated versus unpopulated islands across specified 10-m depth intervals. Probability estimates are derived from the percentage difference in posterior predicted changes in fish biomass at specified depth ranges between unpopulated and populated islands with bathymetric steepness held constant at the study mean value.

**Depth (m)**	**Total fish biomass**	**Primary consumer**	**Planktivore**	**Secondary consumer**	**Piscivore**
0–10	0.74	**1.00**	0.11	**0.77**	**0.85**
10–20	0.74	**1.00**	0.16	**0.77**	**0.84**
20–30	0.74	**1.00**	0.14	**0.77**	0.52

Probability estimates ≥ 0.75 are highlighted in bold.

Supplementary Table 9 Number of stationary point count surveys with zero-count observations of the four trophic groups of reef fish at populated (P) and unpopulated (U) islands, across depth. The proportion of total zero-count observations for each trophic group of the total surveys within each depth category is indicated in brackets.

Depth (m)	Population status	Primary consumer	Planktivore	Secondary consumer	Piscivore
0–10	P	15 (1.2%)	154 (12.4%)	1 (0.1%)	456 (36.7%)
	U	5 (0.6%)	46 (5.7%)	0 (0.0%)	132 (16.5%)
10–20	P	19 (1.7%)	67 (5.9%)	1 (0.1%)	261 (23.0%)
	U	3 (0.3%)	22 (2.3%)	0 (0.0%)	53 (5.7%)
20–30	P	9 (1.1%)	29 (3.5%)	1 (0.1%)	185 (22.4%)
	U	9 (1.5%)	8 (1.4%)	0 (0.0%)	34 (5.8%)

Supplementary Table 10 Estimated median cross-scale variance partitioning (%), standard deviation (SD) and 95% and 75% credible intervals (upper and lower; CIs) for spatial random-effect terms in Bayesian mixed-effects models. SD estimates are derived from model posterior draws and include hurdle model spatial scale variance where applicable (planktivore and piscivore models).

Fish biomass model	Spatial scale	Variance partitioning across scales (%)	Median SD	95% CIs		75% CIs	
Total fish biomass	Site	51.60	0.62	0.59	0.65	0.60	0.63
	Island	20.67	0.25	0.15	0.37	0.19	0.31
	Ecoregion	27.74	0.33	0.05	1.20	0.15	0.66
Primary consumer	Site	52.26	0.77	0.72	0.81	0.74	0.80
	Island	22.43	0.33	0.22	0.48	0.26	0.41
	Ecoregion	25.31	0.37	0.04	1.52	0.15	0.81
Planktivore	Site	25.29	2.57	2.23	2.94	2.37	2.77
	Island	8.33	0.84	0.39	1.51	0.53	1.19
	Ecoregion	66.38	6.73	2.63	16.27	4.01	10.98
Secondary consumer	Site	62.93	0.62	0.60	0.65	0.61	0.64
	Island	19.58	0.19	0.07	0.30	0.13	0.26
	Ecoregion	17.49	0.17	0.01	0.74	0.04	0.40
Piscivore	Site	38.25	2.19	1.97	2.41	2.06	2.31
	Island	19.06	1.09	0.79	1.50	0.90	1.32
	Ecoregion	42.69	2.44	1.23	5.63	1.62	3.92

Supplementary Table 11 Probabilities of differing variation in fish biomass across hierarchical spatial scales: ecoregion, island, and site. Probability estimates are derived from comparing posterior model standard deviations (SD) at each of the hierarchical spatial terms. Probabilities derived from comparing SD across hurdle model components are indicated by HU.

**Fish biomass model**	**Ecoregion > Island**	**Ecoregion > Site**	**Island > Site**
Total fish biomass	0.67	**0.85**	**1.00**
Primary consumer	0.57	**0.86**	**1.00**
Planktivore	**0.95**	0.36	**1.00**
Planktivore (HU)	**1.00**	**0.99**	>**0.99**
Secondary consumer	0.46	**0.96**	**1.00**
Piscivore	0.65	0.74	>**0.99**
Piscivore (HU)	**0.98**	0.71	**1.00**

Probability estimates ≥ 0.75 are highlighted in bold. Probabilities ≥75% of the inverse difference (i.e., SD being less than rather than more than) are underlined.

Supplementary Table 12 Surveyed fish taxa omitted from the study analyses. Reasons for omission include known systematic detectability bias[2–4] (*Carcharhinidae*, *Carangidae*, *Sphyrnidae*), species not typically reef-associated (*Scombridae*, *Chanidae*, *Myliobatidae*), and cryptobenthic eels whose body-size cannot be estimated (*Congridae, Muraenidae*).

Family	Species	Common name	Trophic group	Reason for omission
Carangidae	*Carangoides ferdau*	Blue trevally	Piscivore	Potential overinflation in estimates
	Carangoides orthogrammus	Island trevally	Piscivore	Potential overinflation in estimates
	Caranx ignobilis	Giant trevally	Piscivore	Potential overinflation in estimates
	Caranx lugubris	Black jack	Piscivore	Potential overinflation in estimates
	Caranx melampygus	Bluefin trevally	Piscivore	Potential overinflation in estimates
	Caranx papuensis	Brassy trevally	Piscivore	Potential overinflation in estimates
	Caranx sexfasciatus	Bigeye trevally	Piscivore	Potential overinflation in estimates
	Decapterus macarellus	Mackerel scad	Planktivore	Potential overinflation in estimates
	Elagatis bipinnulata	Rainbow runner	Piscivore	Potential overinflation in estimates
	Scomberoides lysan	Doublespotted queenfish	Piscivore	Potential overinflation in estimates
	Selar crumenophthalmus	Bigeye scad	Planktivore	Potential overinflation in estimates
	Seriola dumerili	Greater amberjack	Piscivore	Potential overinflation in estimates
	Trachinotus baillonii	Smallspotted dart	Piscivore	Potential overinflation in estimates
	Caranx spp.	n/a	Piscivore	Potential overinflation in estimates
Carcharhinidae	*Carcharhinus amblyrhynchos*	Grey reef shark	Piscivore	Potential overinflation in estimates
	Carcharhinus galapagensis	Galapagos shark	Piscivore	Potential overinflation in estimates
	Carcharhinus melanopterus	Blacktip reef shark	Piscivore	Potential overinflation in estimates
	Triaenodon obesus	Whitetip reef shark	Piscivore	Potential overinflation in estimates
Chanidae	*Chanos chanos*	Milkfish	Primary consumer	Non-reef-associated
Congridae	*Congridae* spp.	Conger eel or Garden eel	Piscivore	Cryptobenthic
Muraenidae	*Echidna nebulosa*	Snowflake moray eel	Secondary consumer	Cryptobenthic
	Enchelycore pardalis	Leopard moray eel	Piscivore	Cryptobenthic
	Gymnomuraena zebra	Zebra moray eel	Secondary consumer	Cryptobenthic
	Gymnothorax breedeni	Blackcheek moray eel	Piscivore	Cryptobenthic
	Gymnothorax eurostus	Abbott's moray eel	Secondary consumer	Cryptobenthic
	Gymnothorax flavimarginatus	Yellow-edged moray eel	Piscivore	Cryptobenthic
	Gymnothorax javanicus	Giant moray eel	Piscivore	Cryptobenthic
	Gymnothorax melatremus	Dwarf moray eel	Secondary consumer	Cryptobenthic
	Gymnothorax meleagris	Turkey moray eel	Piscivore	Cryptobenthic
	Gymnothorax steindachneri	Steindachner's moray eel	Piscivore	Cryptobenthic
	Gymnothorax undulatus	Undulated moray eel	Piscivore	Cryptobenthic
	Gymnothorax spp.	Moray eel	Piscivore	Cryptobenthic
Myliobatidae	*Manta birostris*	Giant manta	Planktivore	Non-reef-associated
Scombridae	*Euthynnus affinis*	Kawakawa	Piscivore	Non-reef-associated
	Gymnosarda unicolor	Dogtooth tuna	Piscivore	Non-reef-associated
	Thunnus albacares	Yellowfin tuna	Piscivore	Non-reef-associated
	Scombridae spp.	Striped bonito	Piscivore	Non-reef-associated
Sphyraenidae	*Sphyraena qenie*	Blackfin barracuda	Piscivore	Potential overinflation in estimates

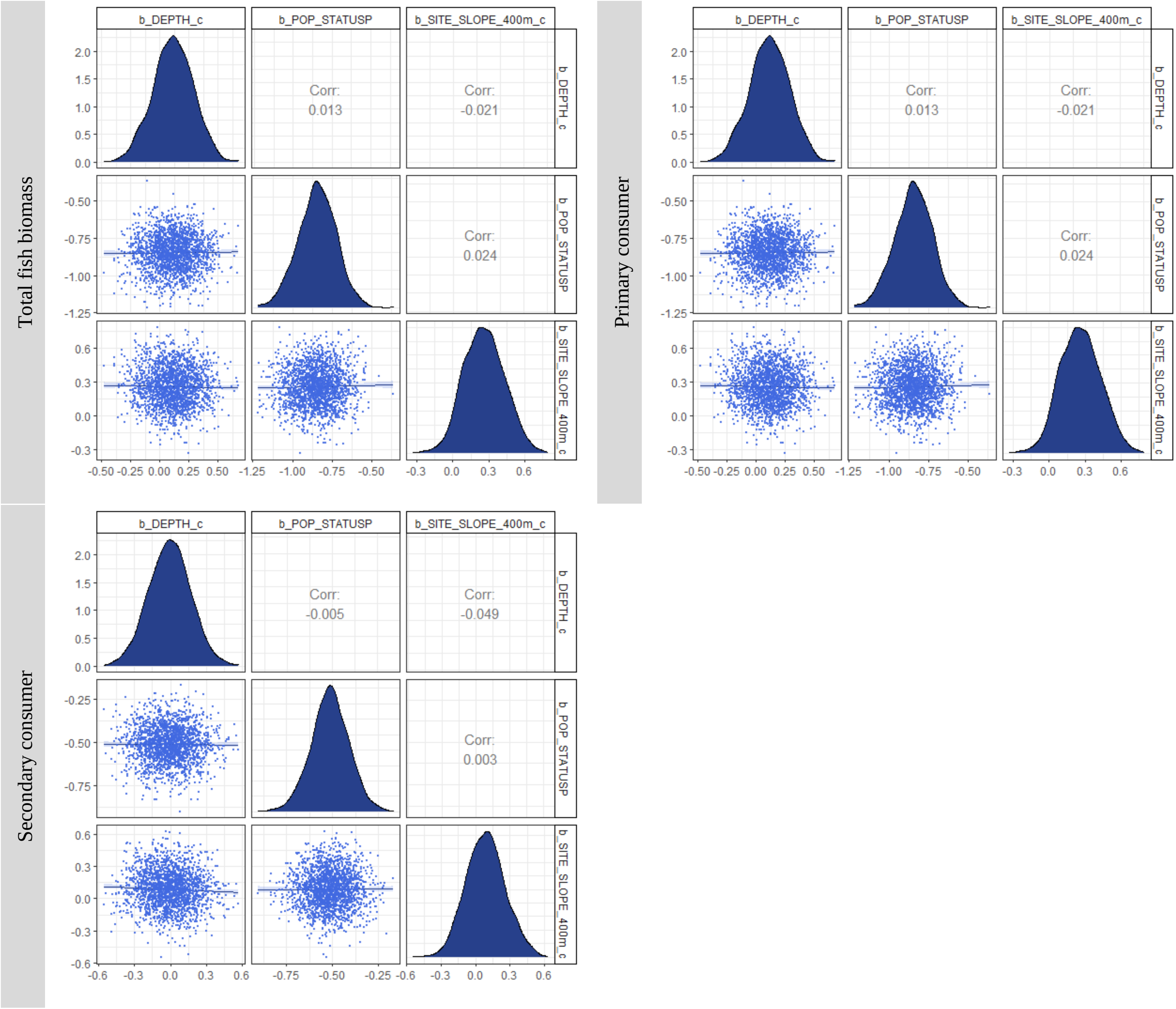
Total fish biomass
Primary consumer
Secondary consumer
b_DEPTH_c
b_POP_STATUSP
b_SITE_SLOPE_400m_c
Corr:
0.013
Corr:
-0.021
Corr:
0.024
Corr:
0.013
Corr:
-0.021
Corr:
0.024
Corr:
-0.005
Corr:
-0.049
Corr:
0.003

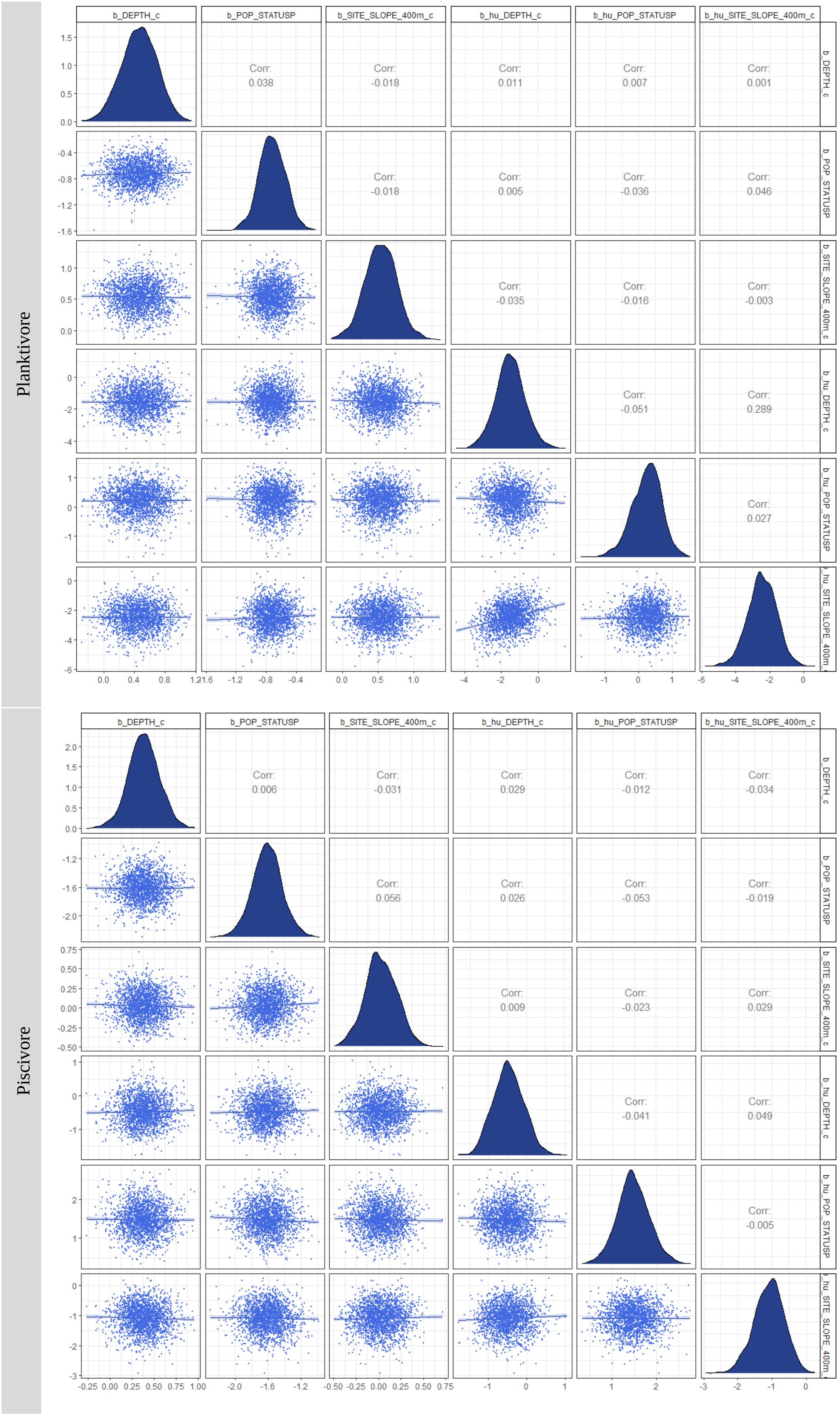

Supplementary Figure 1 Bivariate correlations between biomass models' posterior samples (MCMC draws) of predictor coefficients and Pearson correlation coefficients between paired samples. b_*predictor* relates to model slope coefficients for population-level effects: DEPTH_c (centred and scaled depth effect); POP_STATUSP (human population status); SITE_SLOPE_400m_c (centred and scaled bathymetric steepness effect).

Supplementary information references

1. Gelman, A., Goodrich, B., Gabry, J. & Vehtari, A. R-squared for Bayesian Regression Models. *Am. Stat.* **73**, 307–309 (2019).
2. Kulbicki, M. How the acquired behaviour of commercial reef fishes may influence the results obtained from visual censuses. *J. Exp. Mar. Bio. Ecol.* **222**, 11–30 (1998).
3. Parrish, F. A. & Boland, R. C. Habitat and reef-fish assemblages of banks in the Northwestern Hawaiian Islands. *Mar. Biol.* **144**, 1065–1073 (2004).
4. Williams, I. D. *et al.* Human, oceanographic and habitat drivers of Central and Western Pacific coral reef fish assemblages. *PLoS One* **10**, e0120516 (2015).
