# Supplementary material for: Local human impacts disrupt depth-dependent zonation of tropical reef fish communities
Source: Nat Ecol Evol. 2023 Sep 25;7(11):1844–55. doi: 10.1038/s41559-023-02201-x (PMC10627831; doi:10.1038/s41559-023-02201-x)
Supplement: Supplementary file 2 — Reporting Summary [file 41559_2023_2201_MOESM2_ESM.pdf]

nature portfolio

Corresponding author(s): Laura e. Richardson

Last updated by author(s): 2023/07/04

Reporting Summary

Nature Portfolio wishes to improve the reproducibility of the work that we publish. This form provides structure for consistency and transparency in reporting. For further information on Nature Portfolio policies, see our Editorial Policies and the Editorial Policy Checklist.

Statistics

For all statistical analyses, confirm that the following items are present in the figure legend, table legend, main text, or Methods section.

n/a	Confirmed	
☐	☒	The exact sample size (*n*) for each experimental group/condition, given as a discrete number and unit of measurement
☐	☒	A statement on whether measurements were taken from distinct samples or whether the same sample was measured repeatedly
☐	☒	The statistical test(s) used AND whether they are one- or two-sided<br>*Only common tests should be described solely by name; describe more complex techniques in the Methods section.*
☐	☒	A description of all covariates tested
☐	☒	A description of any assumptions or corrections, such as tests of normality and adjustment for multiple comparisons
☐	☒	A full description of the statistical parameters including central tendency (e.g. means) or other basic estimates (e.g. regression coefficient) AND variation (e.g. standard deviation) or associated estimates of uncertainty (e.g. confidence intervals)
☒	☐	For null hypothesis testing, the test statistic (e.g. *F*, *t*, *r*) with confidence intervals, effect sizes, degrees of freedom and *P* value noted<br>*Give P values as exact values whenever suitable.*
☐	☒	For Bayesian analysis, information on the choice of priors and Markov chain Monte Carlo settings
☐	☒	For hierarchical and complex designs, identification of the appropriate level for tests and full reporting of outcomes
☐	☒	Estimates of effect sizes (e.g. Cohen's *d*, Pearson's *r*), indicating how they were calculated

Our web collection on statistics for biologists contains articles on many of the points above.

Software and code

Policy information about availability of computer code

Data collection	We derived site-level estimates of bathymetric steepness (°) from depth mosaics created from multibeam SONAR, bathymetric LiDAR, and imagery derived depths in ArcGIS Pro v2.7 using the 'Slope' tool (Spatial Analyst).
Data analysis	All analyses were conducted in R 4.2.1. Bayesian hierarchical models were implemented in cmdstanr using brms 2.17.0; probability of covariate effect direction was estimated with bayestestR 0.10.0; model information for querying posterior predictions was extracted with tidybayes 3.0.2; cross-spatial model variance was plotted with TernaryPlot in Ternary 1.2.3; and model fits assessed using r2_bayes in performance 0.9.2. All data and R code used in this study are available at an open-source repository (https://github.com/LauraERichardson/Depth-Fish).

For manuscripts utilizing custom algorithms or software that are central to the research but not yet described in published literature, software must be made available to editors and reviewers. We strongly encourage code deposition in a community repository (e.g. GitHub). See the Nature Portfolio guidelines for submitting code & software for further information.

Data

Policy information about availability of data

All manuscripts must include a data availability statement. This statement should provide the following information, where applicable:

- Accession codes, unique identifiers, or web links for publicly available datasets
- A description of any restrictions on data availability
- For clinical datasets or third party data, please ensure that the statement adheres to our policy

The dataset generated during and/or analysed during the current study are available on Github (https://github.com/LauraERichardson/Depth-Fish).

Research involving human participants, their data, or biological material

Policy information about studies with human participants or human data. See also policy information about sex, gender (identity/presentation), and sexual orientation and race, ethnicity and racism.

Reporting on sex and gender	*Use the terms sex (biological attribute) and gender (shaped by social and cultural circumstances) carefully in order to avoid confusing both terms. Indicate if findings apply to only one sex or gender; describe whether sex and gender were considered in study design; whether sex and/or gender was determined based on self-reporting or assigned and methods used. Provide in the source data disaggregated sex and gender data, where this information has been collected, and if consent has been obtained for sharing of individual-level data; provide overall numbers in this Reporting Summary. Please state if this information has not been collected. Report sex- and gender-based analyses where performed, justify reasons for lack of sex- and gender-based analysis.*
Reporting on race, ethnicity, or other socially relevant groupings	*Please specify the socially constructed or socially relevant categorization variable(s) used in your manuscript and explain why they were used. Please note that such variables should not be used as proxies for other socially constructed/relevant variables (for example, race or ethnicity should not be used as a proxy for socioeconomic status). Provide clear definitions of the relevant terms used, how they were provided (by the participants/respondents, the researchers, or third parties), and the method(s) used to classify people into the different categories (e.g. self-report, census or administrative data, social media data, etc.) Please provide details about how you controlled for confounding variables in your analyses.*
Population characteristics	*Describe the covariate-relevant population characteristics of the human research participants (e.g. age, genotypic information, past and current diagnosis and treatment categories). If you filled out the behavioural & social sciences study design questions and have nothing to add here, write "See above."*
Recruitment	*Describe how participants were recruited. Outline any potential self-selection bias or other biases that may be present and how these are likely to impact results.*
Ethics oversight	*Identify the organization(s) that approved the study protocol.*

Note that full information on the approval of the study protocol must also be provided in the manuscript.

Field-specific reporting

Please select the one below that is the best fit for your research. If you are not sure, read the appropriate sections before making your selection.

☐ Life sciences ☐ Behavioural & social sciences ☒ Ecological, evolutionary & environmental sciences

For a reference copy of the document with all sections, see nature.com/documents/nr-reporting-summary-flat.pdf

Ecological, evolutionary & environmental sciences study design

All studies must disclose on these points even when the disclosure is negative.

Study description	To examine the fish zonation across depths and investigate how humans may impact natural zonation on coral reefs, we used monitoring data from a standardized dataset of underwater visual fish surveys spanning the central and western Pacific (Heenan et al. 2017; https://www.nature.com/articles/sdata2017176). Surveys (n=5525) were carried out at sites around islands and atolls (hereafter 'islands') classified as 'populated' or 'unpopulated' based on unpopulated islands having <50 residents and located >100 km from the nearest larger human settlement using the 2010 US census (www.census.gov/2010census). Of the 35 study islands, 21 were classified as unpopulated (n = 2,321 distinct surveys, across 923 sites) and 14 as populated (n = 3,204 distinct surveys, across 1,330 sites) (Table S1). Islands were also classified by their location within ecoregions: Hawaii Islands; Line Islands; Mariana Islands; Phoenix, Tokelau, Northern Cook Islands; and Samoa Islands. The location of each unique site was pre-selected by randomised stratified design per sampling units of the Pacific RAMP/NCRMP protocol (island, group of small islands, or subsections of larger islands). The target sampling domain was hard-bottom substrate, with sampling effort stratified by reef zone and depth (0–6 m; 6–18 m; 18–30 m).
Research sample	Underwater visual surveys were conducted at distinct sites using the stationary point count method, recording the species, abundance, and body-size of coral-reef fish observed within 15-m diameter cylindrical plots. This is the standard monitoring method

	for the National Oceanic and Atmospheric Administration (NOAA) Pacific Reef Assessment and Monitoring Program (RAMP; 2010-2012) and NOAA's National Coral Reef Monitoring Program (NCRMP; 2013-2019).
Sampling strategy	The number of ecoregions (n=5), islands and atolls (n = 35), sites (n=2253), SPC surveys (n=5525) was chosen to maximize the study sample size based on the availability of standardized monitoring data.
Data collection	he abundance and body-size of all diurnal, non-cryptic reef fishes were estimated using stationary point count (SPC) surveys. At each site, divers conducted simultaneous visual fish counts within 1–4 adjacent, visually-estimated 15-m diameter cylindrical plots, extending from the substrate to the limit of vertical visibility. First, divers compiled lists of all species observed within the survey area over a 5-min period, then counted and estimated the size (total length, TL, to the nearest cm) of listed species present within the cylinder over approximately 30-mins. Full details on SPC survey methods are available in the published data source by Heenan et al. 2017 [https://www.nature.com/articles/sdata2017176].
Timing and spatial scale	Data were collected using underwater visual census stationary point count (SPC) surveys (n=5,525) from 2,253 forereef sites (≤ 30 m depth), conducted at 35 US and US-affiliated islands and atolls in the Pacific Ocean, across 42 degrees (°) of latitude (14°S to 28°N), and 62° of longitude (178° W to 145°E). The data were collected between 2010–2014 for the National Oceanic and Atmospheric Administration (NOAA) Pacific Reef Assessment and Monitoring Program (RAMP; 2010-2012) and NOAA's National Coral Reef Monitoring Program (NCRMP; 2013-2019), conducted by the Ecosystem Sciences Division (ESD) of NOAA's Pacific Islands Fisheries Science Center (PIFSC). Each site was visited a single time and their location was pre-selected by randomised stratified design per sampling units of the Pacific RAMP/NCRMP protocol (island, group of small islands, or subsections of larger islands) (see Heenan et al. 2017 for details; https://www.nature.com/articles/sdata2017176#Sec2). The timing of surveys were determined by the schedule of research expeditions across the region.
Data exclusions	We constrained the dataset to forereef habitat only to remove any possible confounding effects of habitat type on reef fish assemblages. Taxa that are not typically reef-associated were excluded from the analyses, including tuna, bonito, and milkfish (families Chanidae, Myliobatidae, Scombridae; Table S12 in the Supplemental Information). Sixteen species of shark, jack, and barracuda (families Carcharhinidae, Carangidae, Sphyrnidae) were also excluded from the analyses as these highly mobile, large-bodied, roving piscivores are known to be affected by the presence of stationary divers, typically resulting in systematic over-inflation of visual survey density estimates.
Reproducibility	Detailed description of the methods are provided, and all code and data necessary to reproduce the findings (including figures and tables) are freely available on GitHub.
Randomization	Prior to field data collection, the location of survey sites (latitude and longitude coordinates) was selected from sampling strata via a randomized depth-stratified design, with the goal of surveying reefs as widely as possible around and across islands and atolls, on hard-bottom substrate in water shallower than 30m.
Blinding	Blinding was used during data collection by preselecting distinct survey locations via a randomized depth-stratified design (detailed in Heenan et al. 2017; https://www.nature.com/articles/sdata2017176#Sec2).

Did the study involve field work? ☒ Yes ☐ No

Field work, collection and transport

Field conditions	Surveys were conducted when seas were calm and underwater visibility was clear. Long-term mean SST in the region is approximately 27° C (Gove et al. 2013; https://doi.org/10.1371/journal.pone.0061974).
Location	Data were collected from 5,525 surveys on 2,253 forereef sites (≤ 30 m depth) conducted on 35 US and US-affiliated islands and atolls across 42 degrees (°) of latitude (14°S to 28°N), and 62° of longitude (178° W to 145°E).
Access & import/export	Data used are acquired from fishery-independent coral reef surveys as part of the National Oceanic and Atmospheric Administration (NOAA) Pacific Reef Assessment and Monitoring Program (RAMP; 2010-2012) and NOAA's National Coral Reef Monitoring Program (NCRMP; 2013-2019). These data are made publicly available and can be accessed for all NCRMP jurisdictions online. In this instance, the study data was provided on request by Dr Tye Kindinger in NOAA's Pacific Islands Fisheries Science Center.
Disturbance	Only observational data were collected in this study, thus there was minimal disturbance caused.

Reporting for specific materials, systems and methods

We require information from authors about some types of materials, experimental systems and methods used in many studies. Here, indicate whether each material, system or method listed is relevant to your study. If you are not sure if a list item applies to your research, read the appropriate section before selecting a response.

Materials & experimental systems

n/a	Involved in the study
☒	☐ Antibodies
☒	☐ Eukaryotic cell lines
☒	☐ Palaeontology and archaeology
☒	☐ Animals and other organisms
☒	☐ Clinical data
☒	☐ Dual use research of concern
☒	☐ Plants

Methods

n/a	Involved in the study
☒	☐ ChIP-seq
☒	☐ Flow cytometry
☒	☐ MRI-based neuroimaging
